# Jugular Foramen Syndrome: Concurrent Neurological Deficits, Advanced Imaging Findings, Underlying Diagnoses, and Outcomes in 14 Dogs (2016–2024)

**DOI:** 10.1111/jvim.70088

**Published:** 2025-04-29

**Authors:** Megan Madden, Theofanis Liatis, Cesar Llanos, Sumari Dancer, Patricia Alvarez, Sarah Tayler, Alexandros Hardas, Steven De Decker

**Affiliations:** ^1^ Hospital for Small Animals, Royal (Dick) School of Veterinary Studies University of Edinburgh Midlothian UK; ^2^ Department of Clinical Science and Services, Royal Veterinary College University of London Hertfordshire UK; ^3^ Pride Veterinary Referrals Derby UK; ^4^ Department of Pathobiology and Population Sciences, Royal Veterinary College University of London Hertfordshire UK

**Keywords:** accessory, cranial nerve, glossopharyngeal, jugular foramen syndrome, vagus

## Abstract

**Background:**

Jugular foramen syndrome (JFS), dysfunction of cranial nerves (CNs) IX, X, and XI caused by lesions involving the jugular foramen (JF), is rarely reported in dogs.

**Objective:**

Describe presenting complaints, neurologic findings, advanced imaging findings, underlying diagnoses, and outcomes in dogs with JFS.

**Animals:**

Fourteen client‐owned dogs.

**Methods:**

Retrospective, multicenter study of dogs diagnosed with JFS using advanced imaging between 2016 and 2024.

**Results:**

Affected dogs were older (median age, 9.9 years; range, 7.9–14.5 years) and presented with chronic progressive clinical signs (median duration, 135 days; range, 5–720 days). Common presenting complaints included coughing (7/14), retching (6/14), head tilt (5/14), and laryngeal stridor (4/14). Neurologic abnormalities were noted in 11/14 dogs, with CN deficits (10/11), including unilateral laryngeal paralysis (5/10) and tongue atrophy (4/10), being the most common finding. Additional signs included head tilt (7/11) and postural reaction deficits (5/11). Intracranial lesions were identified in 10/14 dogs, with meningioma being the most frequent radiologic or histopathologic diagnosis. In dogs with extracranial lesions (4/14), thyroid carcinoma was common. Median survival time was 218 days (range, 16–477 days).

**Conclusion and Clinical Importance:**

Neoplastic or suspected neoplastic causes of JFS are common and lesions often extend beyond the JF by the time of diagnosis. As such, neurologic deficits in dogs with JFS often reflect involvement of multiple CNs, not limited to CNs IX, X, and XI. Advanced imaging of the head should be considered in dogs with clinical signs consistent with JFS.

**Abbreviations**
JFS: jugular foramen syndrome
JF: jugular foramen
CN: cranial nerve
CT: computed tomography
MRI: magnetic resonance imaging
T2W: T2‐weighted
T1W: T1‐weighted
FLAIR: fluid attenuating inversion recovery
T2*W‐GRE: gradient echo
PNST: peripheral nerve sheath tumor

## 1 Introduction

Jugular foramen syndrome (JFS) describes the combined dysfunction of cranial nerves (CNs) IX (glossopharyngeal nerve), X (vagus nerve), and XI (accessory nerve) caused by lesions affecting their common pathway through the skull [1]. These CNs exit the cranial vault via the jugular foramen (JF) and, together with the internal jugular vein, emerge through the tympano‐occipital fissure [2]. Clinical signs of JFS in humans include dysphonia (CN X), dysphagia (CNs IX and X), loss of taste and sensation in the caudal third of the tongue (CN IX), and shoulder weakness associated with paresis of the sternocleidomastoid and trapezius muscles (CN XI) [1, 3]. However, depending on the extent of the lesion, additional CNs also may be affected (Table 1) [3].

**TABLE 1:** Jugular foramen syndrome and its clinical variants reported in human medicine [3].

Syndrome	Cranial nerve (CN) dysfunction
Vernet syndrome	CNs IX, X, and XI
Jackson syndrome	CNs X, XI, and XII
Collet‐sicard syndrome	CNs IX, X, XI, and XII
Villaret syndrome	CNs IX, X, XI, XII, and ipsilateral Horner syndrome
Tapia syndrome	CNs X and XII
Avellis syndrome	Vocal cord and palatal paralysis with contralateral dissociate hemianesthesia
Schmidt syndrome	A lesion of the vagal and both the bulbar and spinal accessory nuclei

In humans, JFS is most commonly caused by neoplastic masses involving the JF [3], which can be intrinsic (originating from structures within the JF) or extrinsic (arising from surrounding tissues) [4]. Paraganglioma is the most frequently diagnosed primary neoplastic cause of JFS in humans, followed by schwannoma and meningioma [5].

Reports of JFS in dogs are limited to a case report describing a dog with tympanic bulla osteoma [6] and a case series describing the computed tomography (CT) features of five dogs with JFS [7]. Similar to humans, dogs in these reports presented with clinical signs of upper respiratory tract noise and dysphagia, in addition to coughing, retching, gagging, vomiting, hypersalivation, lip smacking, and lethargy [6, 7]. Information regarding the neurologic examination findings in these dogs was not provided. In another case series, imaging findings were consistent with single, intracranial, extra‐axial, and strongly contrast‐enhancing cerebellomedullary mass lesions extending into the JF, with radiological diagnoses of meningioma, peripheral nerve sheath tumor (PNST) or lymphoma [1]. However, histopathological data was not available. Despite recent descriptions of JFS in dogs [6, 7], important knowledge gaps remain, including details of the neurologic examination findings, magnetic resonance imaging (MRI) characteristics, histopathologic diagnoses, and long‐term outcomes for affected dogs. This information is crucial for guiding clinical decision‐making and informing client discussions regarding diagnosis, treatment, and prognosis.

We aimed to describe the neurologic examination and advanced imaging findings in dogs with JFS and to explore underlying causes and long‐term outcomes.

## 2 Materials and Methods

Our study was an observational, multicenter, retrospective study. Ethical approval was obtained by the Royal (Dick) School of Veterinary Studies Veterinary Ethical Review Committee (8.23). Client consent had been obtained previously using institutional consent forms. Electronic records of 2 large veterinary referral hospitals in the United Kingdom between 2016 and 2024 were searched. Inclusion criteria included complete medical records, diagnosis of a lesion affecting the JF based on MRI or CT of the head, and a full neurologic examination performed by a board‐certified neurologist or neurology resident‐in‐training. Clinical data included signalment, duration of clinical signs, presenting complaints, physical, and neurologic examination findings, clinicopathologic, and diagnostic imaging findings, treatment, and outcome. Duration of clinical signs was classified as chronic (> 15 days), subacute (< 15 days), or acute (< 7 days) according to the clinical history. Magnetic resonance imaging was performed under general anesthesia using a high‐field magnet (Intera 1.5T, Philips Healthcare, Amsterdam, Netherlands; or 1.5T, Avanto, Siemens Healthineers, Erlangen, Germany). Computed tomography was performed under general anesthesia or sedation using a 320‐slice (Aquilion ONE Genesis Edition, Canon Medical Systems, Otawara, Japan), 16‐slice (Mx8000 IDT, Philips, Best, the Netherlands) or 64‐slice (Somatom Definition AS, Siemens AG, Erlangen, Germany) scanner. All imaging studies were retrospectively reviewed by both a board‐certified veterinary radiologist and resident‐in‐training. Reviewers were not completely blinded to the imaging diagnosis before reviewing the images because some of the cases previously had presented at their own institution. Images were reviewed using digital imaging and communications in medicine (DICOM) viewer (Osirix Imaging Software, version 10.11, Bernex, Switzerland; Horos Project, version 2.2.0, horosproject.org, ExploreDTI, version 4.8.6, Utrecht) or online imaging processing computer software (eUnity v7.1.204, Mach7 Technologies, Waterloo, Ontario, Canada). Imaging criteria were defined (Table S1) and recorded for each imaging study by each reviewer independently. In cases where discrepancies were identified between reviewers, the images were reviewed by a third person (a board‐certified neurologist). The origin of the JF lesion for each case was defined as intracranial or extracranial. The extent of the lesion was classified according to whether it occupied the intracranial, intraforaminal, intrafissural, or extracranial compartment. A presumptive diagnosis of meningioma was based on imaging features such as a single, T1‐weighted (T1W) hypo‐ to isointense, T2‐weighted (T2W)/T2‐fluid attenuating inversion recovery (FLAIR) hyperintense, strongly contrast‐enhancing, extra‐axial mass lesion with dural tail sign [8, 9, 10]. Descriptive statistics were performed using Minitab 20 (Minitab LLC).

## 3 Results

### 3.1 Signalment and Presenting Complaints

Fourteen dogs met the inclusion criteria. Median age was 9.95 years (range, 7.9–14.5 years) and median body weight was 17.65 kg (range, 9.5–32.8 kg). There were 4/14 neutered males and 10/14 females (8/10 spayed). Breeds included three Staffordshire Bull Terriers, two West Highland White Terriers, and one each of: Boxer, French Bulldog, Irish Setter, Labrador Retriever, Lhasa Apso, Soft‐Coated Wheaten Terrier, Springer Spaniel, Sussex Spaniel, and Pug cross. Onset of clinical signs was chronic in 11/14 dogs and acute in 3/14 dogs. The median duration of clinical signs before presentation was 135 days (range, 5–720 days). All dogs were referred with two or more presenting complaints. The most common presenting complaints were coughing (7/14), retching (6/14; Video 1), head tilt (5/14), regurgitation (4/14), ataxia (4/14), sneezing (3/14), and lethargy (3/14; Table S2). Two dogs had known concurrent medical conditions at the time of presentation, including hyperadrenocorticism (1/2) and intervertebral disc disease (1/2).

**VIDEO 1:** Characteristic semiology in a dog with jugular foramen syndrome showing laryngeal stridor and retching. Video content can be viewed at https://onlinelibrary.wiley.com/doi/10.1111/jvim.70088 Video content can be viewed at https://onlinelibrary.wiley.com/doi/10.1111/jvim.70088

### 3.2 Clinical Findings

Abnormal physical examination findings were present in 8/14 dogs, including coughing or stridor (4/8; Video 1), a palpable soft tissue mass in the ventral cranial cervical region (2/8), unilateral saliva accumulation in the oral cavity (2/8), gagging and retching after the tracheal pinch test (1/8), unilateral xeromycteria (2/8), pain on opening the mouth (1/8), corneal ulceration (1/8), and otitis externa (1/8; Table S2).

**FIGURE 1:**
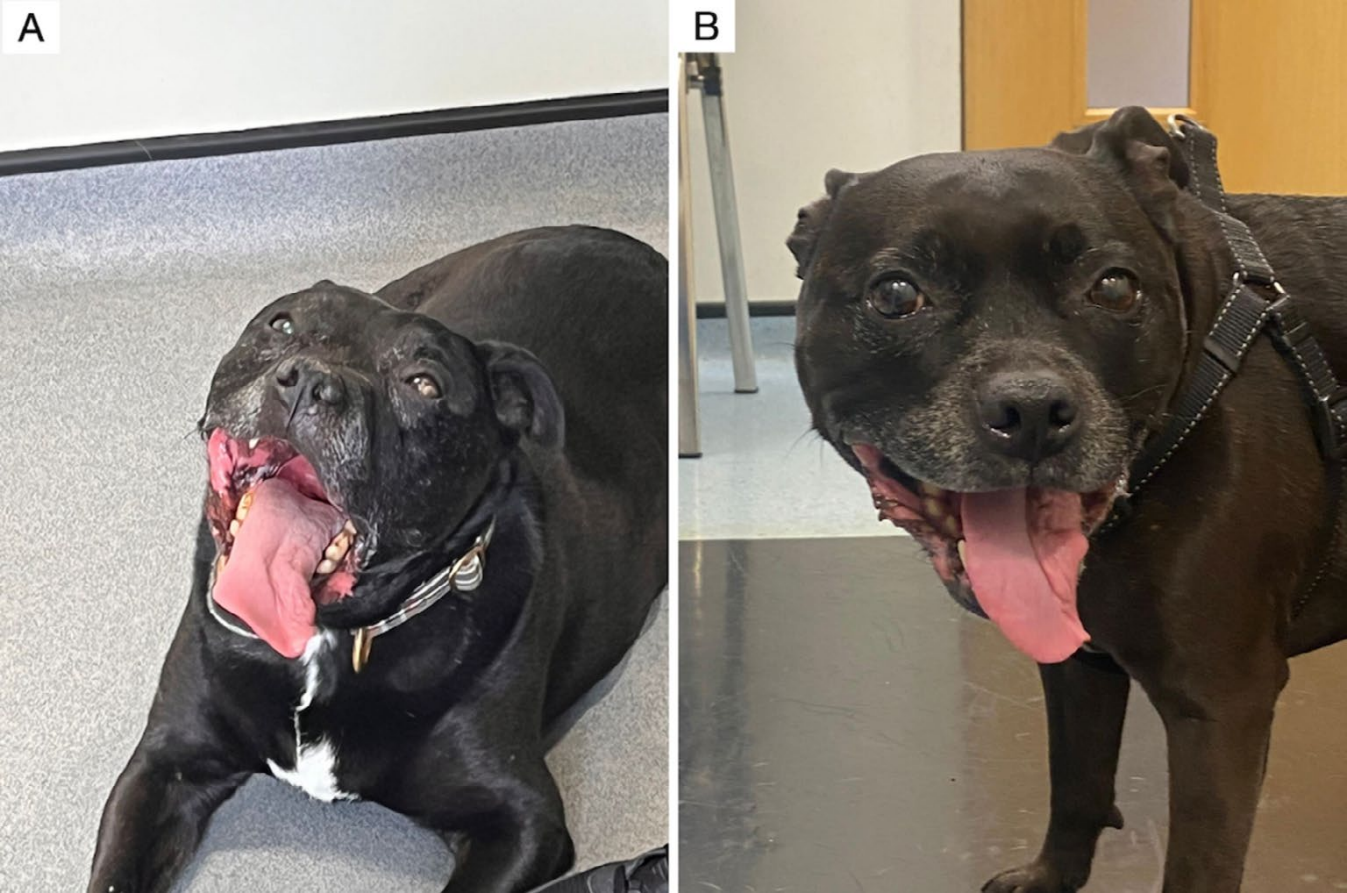
Unilateral left‐sided tongue atrophy in two dogs (Case 10 [1A] and Case 14 [1B]) with JFS, both diagnosed with a left‐sided extra‐axial plaque‐like cerebellopontine angle mass (suspected meningioma).

The neurologic examination was abnormal in 11/14 dogs (Table S3). The most common neurologic examination findings included: CN deficits (10/11), head tilt (7/11) and postural reaction deficits (5/11). Unilateral laryngeal paralysis (5/10) and unilateral tongue atrophy (4/10, Figure 1) were the most common CN deficits. In 1 dog, laryngeal paralysis had been diagnosed and treated surgically 2 years before presentation, when a CT study of the head was reported to be normal. In the remaining 4/5 cases, airway examination under general anesthesia was required for diagnosis. Laryngeal stridor (4/5) or dysphonia (1/5) was reported in all dogs with confirmed laryngeal paralysis. For two additional dogs with clinical signs of laryngeal stridor or dysphonia, it was not reported whether an airway examination had been performed. Neuroanatomic localizations included a cranial polyneuropathy, either central (i.e., affecting the cell bodies in the brainstem or portions of the relevant CNs before exiting the meninges) or peripheral (i.e., from their exit from the foramen to their target tissues; 5/11), central (2/11), or peripheral (1/11) vestibular system, or multifocal (3/11). Two dogs with multifocal neurolocalizations had neurologic deficits consistent with CN XII (Case 13) or peripheral vestibular system (Case 2) involvement, along with a T3‐L3 myelopathy caused by intervertebral disc disease (Case 13) or degenerative myelopathy (Case 2), respectively, diagnosed during investigations and unrelated to their presenting clinical signs. One dog (Case 14) with a cranial polyneuropathy had chronic, concurrent T3‐L3 myelopathy associated with a previous thoracolumbar corpectomy for treatment of an intervertebral disc protrusion. Lateralization of neurologic signs related to JFS was present in all dogs with abnormal neurological examination findings (left 6/11; right 5/11). In 3 dogs with normal neurologic examination, neurolocalization to CNs IX or X or both was considered to be caused by retching (2/3), stridor or dysphonia (2/3), and regurgitation (1/3).

### 3.3 Clinicopathological Findings

Hematology and serum biochemistry were performed in 12/14 dogs and identified mild non‐specific findings (Table S4). Cerebrospinal fluid analysis was performed in 2 dogs with abnormalities detected in both, including neutrophilic pleocytosis (Case 4) and albuminocytologic dissociation (Case 11). Cytology of fine‐needle aspirates from two dogs with extracranial masses was consistent with epithelial carcinoma (Case 3) and thyroid neoplasia (Case 5).

### 3.4 Advanced Imaging Findings

Four dogs underwent MRI only, 6/14 dogs underwent CT imaging only, and 4/14 dogs had both imaging modalities performed. In cases where both CT and MRI were performed, in 3/4 these procedures were not performed on the same day (median interval, 12 days; range, 8–23 days). Lesions were determined to originate from intracranial structures in 10/14 dogs and extracranial structures in 4/14 dogs.

#### 3.4.1 MRI Findings

Magnetic resonance imaging was performed in 8/14 dogs (Table S5) including the head (7/8) and the head and thoracolumbar vertebral column (1/8). Involvement of the JF and CNs IX, X, and XI was identified in all cases as an extension of the primary lesion through the JF. Single lesions were identified in all cases, originating from the intracranial space in 7/8 dogs and the extracranial space in 1/8 dog.

**FIGURE 2:**
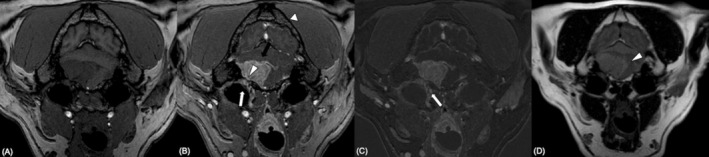
Confirmed mixed/transitional grade 1 meningioma (Case 8). Magnetic resonance imaging study of the head at the level of the tympanic bullae. Transverse 3D T1W spoiled Gradient Echo pre‐contrast (A), post‐contrast (B), subtraction (C), and 2D T2W‐FLAIR TSE (D). Single, ovoid, extra‐axial, homogeneous contrast‐enhancing mass causing widening of the JF (B; arrowhead), tympano‐occipital fissure (C; arrow) and indentation of the dorsal margin of the tympanic bulla (B; arrow). The mass is producing moderate mass effect and mild perilesional vasogenic edema (D: Arrowhead).

In dogs with intracranial lesions, widening of the JF (Figure 2B) was detected in all dogs. Other osseous changes included widening of the tympano‐occipital fissure (6/7; Figure 2C), widening of the hypoglossal canal (2/7), and indentation of the dorsal margin of the tympanic bulla (2/7; Figure 2B). Soft tissue changes included ipsilateral atrophy of the tongue (1/7), temporalis muscle (1/7), or caudal belly of the digastricus muscle (2/7), and extension of contrast enhancement through the hypoglossal canal (3/7). Assessment of all muscle groups innervated by CN XI (trapezius, cleidocephalicus, sternocephalicus, omotransverse) was not possible in all 7 dogs because the neck and thorax were not included in the MRI studies. However, ipsilateral atrophy of the mastoid and occipital part of the sternocephalicus muscle was detected in one dog. Imaging findings involving the neural structures included mass effect (7/7; Figure 2D), perilesional edema (4/7) and obstructive hydrocephalus characterized by generalized ventriculomegaly, flattening of the cortical sulci, dilatation of the olfactory recesses, and periventricular edema (1/7). Additional CN involvement (other than CNs IX, X, and XI) was detected in 6/7 dogs, including ipsilateral CN V contrast enhancement (2/7) and CN VII (5/7), CN VIII (5/7), and CN XII (3/7) thickening and contrast enhancement. All intra‐cranial lesions were focal, extra‐axial, and well‐defined mass lesions within the caudal cranial fossa (Figure 2). Signal intensity of the lesions (relative to the surrounding gray matter) was T2W hyperintense (7/7), T2‐FLAIR hyperintense (6/7), and T1W hypo‐ to isointense (6/7). All lesions were strongly contrast‐enhancing, with a homogeneous (4/7), heterogenous (2/7), or ring‐enhancing pattern (1/7) of contrast uptake. Dural tail sign was present in all dogs. Lesion distribution involved the intracranial, intraforaminal, and intrafissural compartments in all dogs.

**FIGURE 3:**
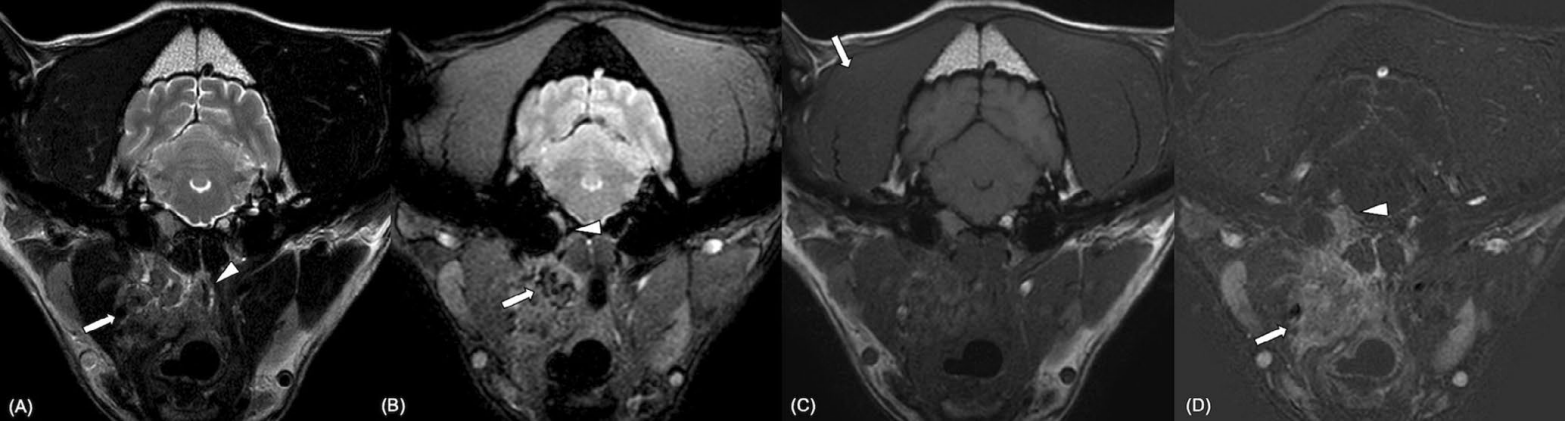
Thyroid carcinoma (cytological diagnosis; Case 3). Magnetic resonance imaging study of the head at the level of the tympanic bullae. Transverse 2D T2W TSE (A), 2D T2*W Gradient Echo (B), 2D T1W TSE pre‐contrast and 2D T1W TSE post‐contrast subtraction (D). Heterogeneous contrast‐enhancing mass surrounding the right carotid artery (A, D: Arrow). It has a salt and pepper appearance with numerous T2*W foci of susceptibility artifact (B; arrow). It is producing nasopharyngeal compression (A: Arrowhead), widening of the tympano‐occipital (B; arrowhead), and meningeal enhancement (D; arrowhead). Mild right‐sided temporalis muscle atrophy is also identified (C; arrow).

In the dog with an extracranial lesion (Case 3, Figure 3) widening of the tympano‐occipital fissure (Figure 3B) and condylar canal, but not of the JF, was present. Soft tissue changes included nasopharyngeal compression (Figure 3A), ipsilateral temporalis muscle atrophy (Figure 3C), incorporation of the internal carotid artery (Figure 3A,D) and involvement of the longus capitis muscle. Meningeal contrast enhancement (Figure 3D) and asymmetrical contrast enhancement of the ipsilateral trigeminal nerve were present. Lesion signal intensity was heterogeneously T2W hyperintense (Figure 3A), T1W iso‐to‐hyperintense (Figure 3C), with multiple small foci of susceptibility artifact on T2*W‐gradient echo (T2*W‐GRE) sequences (salt and pepper appearance; Figure 3B) with marked, heterogeneous contrast enhancement (Figure 3D). The lesion extended from the extracranial structures to occupy the intrafissural, intraforaminal, and intracranial compartments. Involvement of the intracranial compartment was determined by the presence of meningeal contrast enhancement adjacent to the JF.

#### 3.4.2 CT Findings

A CT study of the head was performed in 10/14 dogs (Table S6). Additional study regions included the neck (6/10), thorax (8/10), thoracic limbs (1/10), thoracolumbar vertebral column (1/10), and abdomen (3/10). Involvement of the JF (and thus CNs IX, X, and XI) was confirmed in all dogs as extension of soft tissue‐attenuating and contrast‐enhancing material through the JF. The origin of the lesions was intracranial in 6/10 dogs and extracranial in 4/10 dogs.

**FIGURE 4:**
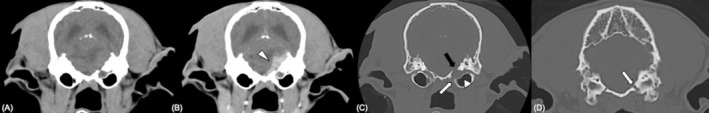
Suspected meningioma (Case 1). Computed tomographic images of the head at the level of the tympanic bullae (A–C) and hypoglossal canal (D). Transverse soft tissue algorithm pre‐contrast (A), post‐contrast (B) and bone algorithm (C and D). Plaque‐like, extra‐axial soft tissue attenuating, homogeneous contrast enhancing mass (B; arrowhead) producing widening of the JF (C; black arrow), tympano‐occipital fissure (C; white arrow) and indentation of the dorsal margin of the tympanic bulla (C; arrowhead). There is extension of the mass into the JF and tympano‐occipital fissure. (D) Widening of the hypoglossal canal (arrow).

**FIGURE 5:**
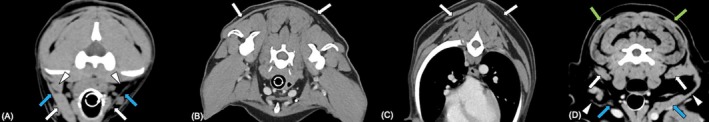
Computed tomographic images of the head (A) and thorax at different levels (B and C) in Case 1. These images illustrate left‐sided muscle atrophy affecting the mastoid part of the sternocephalicus muscle (A; white arrows), mastoid part of the cleidocephalicus muscle (A, arrowheads), occipital part of the sternocephalicus (A; blue arrows), cervical part of the trapezius muscle (B; arrows) and thoracic part of the trapezius muscle (C; arrows). Computed tomographic image of the neck in Case 11 (D) demonstrating right‐sided atrophy of the omotransverse muscle (white arrows), sternocephalicus muscle (blue arrows), mastoid part of the cleidocephalicus muscle (arrowheads) and cervical part of the trapezius muscle (green arrows).

In dogs with intracranial masses, widening of the JF (Figure 4C) was present in 5/6 dogs. Additional changes affecting the osseous structures included widening of the tympano‐occipital fissure (4/6; Figure 4C), indentation of the dorsal margin of the tympanic bulla (2/6; Figure 4C), widening of the hypoglossal canal (4/6; Figure 4D), sclerosis of the petrous temporal bone (1/6) or basioccipital bone (1/6), hyperostosis of the petrous temporal bone (2/6), and thinning of the petrous temporal bone (1/6). Ipsilateral atrophy of the cleidocephalicus and sternocephalicus muscles was present in all 6 dogs (Figure 5A). Atrophy of the trapezius muscle was detected in 4/6 dogs (Figure 5B,C). In the remaining two dogs, it was not possible to assess this muscle because the relevant regions were not included in the imaging study. Although assessment of the omotransverse muscle was possible in all six dogs, ipsilateral atrophy only was detected in 2/6 dogs and was reported to be normal in the remaining dogs (Figure 5D). Laryngeal muscle atrophy, indicating dysfunction of the recurrent laryngeal nerve (branch of the vagus nerve), was present in 3/6 dogs. Atrophy of other muscle groups included the tongue (1/6), indicating hypoglossal nerve (CN XII) involvement, and the caudal belly of the digastricus (1/6), indicating facial nerve (CN VII) involvement. Extension of contrast enhancement through the hypoglossal canal (2/6) and carotid canal (1/6) was also detected. All intracranial mass lesions were described as focal, extra‐axial, and well‐defined. All lesions were soft tissue‐attenuating with strong heterogeneous (3/6) or homogeneous (3/6) contrast enhancement. A fluid‐attenuating component within the lesion was present in 1/6 dog (Case 12). Mass effect was detected in all dogs; MRI studies were available for two of these dogs confirming this finding. Dural tail sign was appreciated in 1/6 dog (Case 1). Perilesional edema was detected in 1/6 dog (Case 1) but was not present in two dogs in which perilesional edema was detected on subsequent MRI studies (Cases 11 and 12). Lesion distribution involved the intracranial, intraforaminal, and intrafissural compartments in all dogs.

**FIGURE 6:**
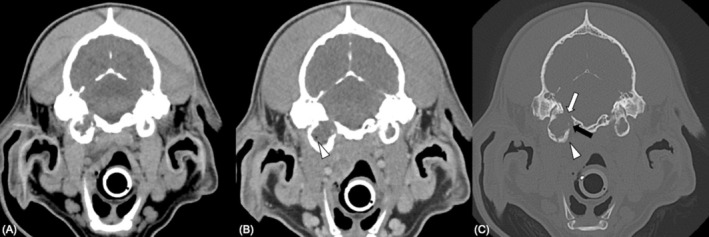
Histologically confirmed cholesteatoma, para‐aural abscess, otitis media and interna (Case 6). Computed tomographic images of the head at the level of the tympanic bullae (A–C). Transverse soft tissue algorithm pre‐contrast (A), post‐contrast (B) and bone algorithm (C). The right middle ear contains a large amount of hypoattenuating, nonenhancing material, and gas (B; arrow). There is widening of the JF (C; white arrow), tympano‐occipital fissure (C; black arrow) and thickening and lysis of the tympanic bulla wall (C; arrowhead).

In dogs with extracranial masses, widening of the JF (Figure 6C) was present in all dogs. Additional changes affecting the bony structures included widening of the tympano‐occipital fissure (3/4; Figure 6C), indentation of the dorsal margin of the tympanic bulla (2/4), widening of the hypoglossal canal (3/4), widening of the condylar canal (1/4), osteolysis of the petrous portion of the temporal bone (1/4), thinning of the tympanic portion of the temporal bone (1/4), osteolysis of the tympanic bulla (2/4) and inner ear structures (1/4; Figure 6C), osteolysis of the petro‐occipital canal (2/4), and widening of the petro‐occipital and carotid canal (1/4). Ipsilateral atrophy of the trapezius, cleidocephalicus, and sternocephalicus muscle groups was present in 2/4 dogs (Cases 3 and 13). The omotransverse muscle group was unaffected in all dogs. Unilateral atrophy of the temporalis muscle was detected in 1/4 dog, indicating CN V involvement. Additional soft tissue findings included invasion of the lesion into the longus capitis muscles (1/4), nasopharyngeal compression (3/4), incorporation of the internal carotid artery (3/4), extension into the carotid canal (1/4), otitis media and externa (1/4; Figure 6), laryngeal displacement (1/4), involvement of the digastricus muscle (2/4) and sternohyoid muscle (1/4) of the larynx, and contrast enhancement extending into the hypoglossal canal (3/4), and carotid canal (2/4). All lesions were soft tissue‐attenuating with strong heterogeneous (3/4) or mildly ring‐enhancing (1/4) contrast uptake. Mass effect on the neural structures was not detected in any of the dogs with extracranial lesions, but extension of soft tissue‐attenuating and contrast‐enhancing material into the intracranial space was present in two dogs. Lesion distribution involved the extracranial, intrafissural, and intraforaminal compartments in one dog, and the extracranial, intrafissural, intraforaminal, and intracranial compartments in three dogs. For dogs in which both MRI and CT studies were available (4/14), lesion distribution differed in one dog (Case 3) because of the inability to detect intracranial extension of the lesion on the CT study. In that dog, the CT and MRI were performed on the same day.

### 3.5 Diagnoses

The most common radiologic diagnosis for dogs with an intracranial lesion was meningioma (10/10; Figures 2 and 4); one of them (Case 8) was histopathologically confirmed as mixed or transitional grade I meningioma (Figure 2). Lesion morphology based on imaging was plaque‐like (7/10), round or ovoid (2/10) or cystic (1/10). In dogs with extracranial lesions, the most common diagnosis was thyroid carcinoma (Cases 3 and 5; 1 cytologically and 1 histopathologically confirmed; Figure 3), followed by suspected carotid body paraganglioma (Case 13) and para‐aural abscess, cholesteatoma, and otitis media and interna (Case 6; histopathologically confirmed; Figure 6).

### 3.6 Treatment and Outcome

Detailed information regarding treatment and outcome is provided in Table S7. Treatment in dogs with intracranial lesions consisted of palliative treatment (corticosteroids) in four dogs, radiation therapy (along with corticosteroids and analgesia) in three dogs, and chemotherapy (hydroxyurea) in one dog. The remaining two dogs were euthanized at the time of diagnosis. In dogs with extracranial lesions, three underwent treatment and one was euthanized immediately after diagnostic investigations. Treatment for thyroid carcinoma consisted of palliative treatment (meloxicam) in one dog and a combination of radiation therapy, imatinib, and meloxicam in the other. In one dog with a para‐aural abscess, cholesteatoma, and otitis media and interna, treatment consisted of a total ear canal ablation and lateral bulla osteotomy, antibiotics, and analgesia. Follow‐up information was available in 7/14 dogs (five with intracranial and two with extracranial lesions), with a median follow‐up time of 218 days (range, 16–477 days). Four dogs were lost to follow‐up, and three dogs were euthanized at the time of diagnosis. For dogs with intracranial lesions, a median survival time of 218 days (range, 16–430 days) was reported. Two dogs with intracranial lesions were euthanized within the study period; one dog was lost to follow‐up, and two dogs (Cases 11 and 12) were still alive. Clinical signs associated with JFS (e.g., retching, gagging) persisted in these two dogs despite treatment (definitive radiation therapy in both cases). Follow‐up information was available for two dogs with extracranial lesions. Both dogs were alive at last follow‐up (211 and 477 days, respectively), with Case 6 (para‐aural abscess, cholesteatoma, otitis media, and interna) reported to be neurologically normal after treatment.

## 4 Discussion

In our study, dogs with JFS were older at presentation with a chronic progressive history of nonspecific clinical signs (e.g., retching, coughing). We found that neurologic examination findings were frequently abnormal in dogs with JFS and were not restricted to CN IX, X, and XI dysfunction, with additional CN involvement, head tilt, or postural reaction deficits being commonly detected. The most common causes of JFS in this population of dogs were intracranial lesions, with meningioma being the most common radiologic or histopathologic diagnosis. Extracranial neoplastic causes of JFS included thyroid or carotid body neoplasia.

Neoplasia was the most common diagnostic imaging or histopathologic diagnosis in this cohort of dogs, consistent with their older age and chronicity of clinical signs. A similar age and duration of clinical signs were reported in a recent study [7], with neoplastic causes prioritized for all dogs based on the described CT findings. Similar to human patients with JFS and previous reports of dogs [7], coughing, and retching were the most common presenting complaints [3, 7]. Although few dogs were referred for neurologic signs, abnormal neurologic findings were common in our study population. However, surprisingly, deficits associated with CNs IX, X, and XI (i.e., those traversing the JF) rarely were detected on neurologic examination alone, suggesting that neurologic examination has low sensitivity for dysfunction of these CNs in dogs. For example, three dogs (Cases 3, 7, and 9) with masses occupying the JF had completely normal neurologic examinations. This observation emphasizes the importance of not excluding dysfunction of CNs IX, X, and XI based solely on a normal neurologic examination, particularly in dogs with a history of coughing, dysphonia or stridor, dysphagia, retching, or gagging.

In humans, assessment of the gag reflex is not commonly performed unless brainstem impairment is suspected, because it can cause unnecessary discomfort. Furthermore, the gag reflex can be absent in up to one third of healthy people and is known to weaken with age [11]. Alternative methods of assessing CN IX and X motor function in humans include cough reflex testing [12], maximal phonation time, and indirect or direct laryngoscopy during respiration and phonation [13]. These tests would not be appropriate in an awake dog, and it is unlikely that more objective means of testing the gag reflex will become feasible in this species. Airway examination under sedation or at the time of induction for general anesthesia should be considered in dogs with clinical signs compatible with JFS to evaluate CN X involvement. Because of the retrospective nature of our study, airway examinations were not performed in all dogs presenting with respiratory stridor or coughing. As such, the frequency of laryngeal paralysis may have been underestimated.

Functional impairment of CN XI in humans is identified by assessing trapezius and sternocleidomastoid strength and mass, by asking the patient to turn the head to each side and to shrug the shoulders against the examiner's resistance [14]. Lateral scapula winging or dropped shoulder, lateral displacement of the scapula caused by unopposed trapezius muscle contraction, is another clinical sign of CN XI impairment [14]. Dropped shoulder was not reported as a clinical finding affecting the dogs in our study, but this abnormality may not have been specifically assessed during their examinations. Although a dropped shoulder was reported as an imaging feature in 4/5 dogs in a previous study [1], the relevance of this finding was questionable given positional variation of the patients during CT. In humans, a dropped shoulder is a clinical finding and not an imaging diagnosis. Careful attention to the symmetry of the scapulae and cervical musculature should be made during the physical or neurological examination of dogs presenting with signs consistent with JFS.

In contrast to previous studies [6, 7], we found that neurologic deficits were common in dogs with JFS. This difference may be explained by the fact that complete neurologic assessment either was not reported or not performed in the previous studies. Importantly, in our study, neurologic deficits were not restricted to the CNs traversing the JF, with many dogs presenting with vestibular signs or other CN deficits because of the extent of pathology (affecting other foramina, the brainstem or both) at the time of presentation. After unilateral laryngeal paralysis, unilateral tongue atrophy was the most frequently detected CN abnormality in dogs with JFS. Because of the proximity of the JF to the hypoglossal canal within the intracranial cavity, and the proximity of the tympanooccipital fissure to the emergence of the hypoglossal canal just rostral to the ventral condyloid fossa, local intra‐ or extracranial extension of a JF mass could lead to CN XII deficits [1, 15, 16]. Together, the combined clinical and diagnostic imaging findings of CNs IX, X, XI, and XII dysfunction are termed “Collet‐Sicard syndrome” [3]. Based on a literature review, Collet‐Sicard syndrome has not been reported in dogs. We identified four dogs with Collet‐Sicard syndrome in our study. In three of these dogs, diagnostic imaging features were consistent with a plaque‐like meningioma. Plaque‐like meningiomas have a predilection for the parasellar or basilar region in dogs [8]. Given the infiltrative nature of plaque‐like meningiomas along dural planes, multiple CN involvement is to be anticipated in the basilar region. Surprisingly, CT or MRI findings consistent with unilateral tongue atrophy only were detected in one dog, despite it being a clinical finding in four dogs. The reason for this discrepancy is unclear given the superior soft tissue detail provided by advanced imaging. Studies investigating the sensitivity of advanced imaging modalities for detecting tongue atrophy in dogs have not been conducted, and it is possible that subjective assessment alone is not particularly sensitive to this clinical finding. Furthermore, the retrospective nature of our study meant that diagnostic imaging protocols were not specifically designed to assess this feature. The influence of the endotracheal tube on tongue position also may be a factor that complicates the interpretation of tongue atrophy on advanced imaging. Establishing objective methods for assessing tongue atrophy, such as calculating area or volume by means of user‐defined regions of interest, could be advantageous for future studies.

After CN deficits, a head tilt was the second most common neurologic finding, identified in seven dogs, six of which had a histologic or radiologic diagnosis of meningioma. In the remaining dog, vestibular signs were attributed to a diagnosis of a cholesteatoma, otitis media, and otitis interna. Magnetic resonance imaging studies were available for 4/6 dogs with head tilt and a histologic or radiologic diagnosis of meningioma. In these studies, the mass extended to the level of the emergence of CN VIII from the brainstem, and thickening or contrast enhancement of CN VIII was visible in all dogs. Given the slow‐growing nature of meningiomas, involvement of other CNs (excluding CNs IX, X, and XI) in dogs with JFS likely results from delayed diagnosis. By the time advanced imaging is performed, the lesion often has spread along meningeal planes to affect additional CNs at their exit from the brainstem. The difficulty in detecting CN IX, X, and XI involvement during routine neurologic examination may lead to misdiagnosis and missed early treatment opportunities. True JFS (restricted to CNs IX, X, and XI involvement) appears to be rare, with only three dogs in our study presenting with clinical signs restricted to involvement of CN IX or X or both, all with normal neurologic examinations. Thus, advanced imaging of the head should be considered even when the neurologic examination appears normal in dogs with clinical signs attributable to JFS.

In our dogs, JF lesions were typically single, intracranial, extra‐axial, and strongly contrast‐enhancing, resembling previous reports [7]. Meningiomas were the most common radiologic diagnosis, confirmed histopathologically in one dog. This finding contrasts with humans, where jugular paragangliomas (glomus jugulare tumors) are the most frequent primary neoplastic cause, followed by schwannomas and meningiomas [5]. In humans, surgical treatment for jugular foraminal masses permits a histologic diagnosis in most cases. Because of the difficulty in accessing the skull base within the caudal fossa, surgery is rarely, if ever, performed in dogs with JF lesions, making necropsy examination necessary for confirmation. Although imaging features in our cases were consistent with meningiomas, considerable overlap exists in imaging features for paragangliomas, schwannomas, and meningiomas in the human medical literature [4, 17, 18, 19, 20, 21, 22]. For these reasons, paragangliomas or schwannomas cannot be fully excluded. Although rare in human medicine, an inflammatory pseudotumor is a possible differential diagnosis for a JF mass [23].

Finally, we identified a previously undescribed population of dogs with JFS in which pathology originated from the extracranial structures. The most common extracranial cause was thyroid carcinoma. Carcinomatous involvement of the JF has been reported in humans caused by primary (nasopharyngeal [24], salivary gland [25]), and metastatic skull base lesions [26]. Two dogs with extracranial pathology had palpable soft tissue masses in the ventral cervical or retropharyngeal region, emphasizing the importance of careful palpation of this area during the physical examination of dogs presenting with clinical signs consistent with JFS. Para‐aural abscess, cholesteatoma, and otitis media and interna described here to cause JFS in a dog have not been reported previously. This diagnosis [27] and middle ear adenocarcinoma [28] previously have been described to cause JFS in cats.

In our study, computed tomography was more sensitive to osseous changes and provided better assessment of the trapezius, cleidocephalicus, sternocephalicus, and omotransverse muscles because the neck and thorax were more commonly included in CT studies compared with MRI. Although CT reliably detected mass effect in dogs with intracranial masses, it was less sensitive to parenchymal and CN pathology. This finding is likely a consequence of beam hardening artifact precluding reliable assessment of CNs and signal changes in this area [29]. Determining the extent of intracranial pathology with MRI can be important to inform treatment options. However, these benefits must be weighed against the availability of MRI, procedure duration, anesthetic risk, and cost. Although CT could not detect subtle thickening or contrast enhancement of individual CNs, involvement of these CNs in most cases could be inferred from extension of contrast‐enhancing masses along the intracranial cavity to the level of the relevant foramina, or by the presence of asymmetrical widening of the foramina. Although MRI better defined the extent of CN involvement along the brainstem, it rarely identified CN involvement that was not apparent from the neurologic examination. In general, in dogs in which both MRI and CT were available, there was fair agreement regarding the extent of spread of the lesion from the intracranial space into the JF, tympano‐occipital fissure, and extracranial space, or vice versa. Thus, if MRI is not feasible, CT of the head along with a thorough neurologic examination is likely to provide a reliable diagnosis of a JF lesion and determine the extent of the lesion. Additionally, CT enables more rapid assessment of other regions, allowing exclusion of other medical conditions that cause overlapping clinical signs with JFS and the detection of metastasis, given the high incidence of neoplastic causes.

Limitations of our study include its retrospective nature, lack of follow‐up data for some cases, variability in treatment protocols, few cases of histopathologically confirmed diagnoses, and few cases with both MRI and CT.

In conclusion, in dogs with JFS, lesions involving the JF often are not detected until late in the disease process, at a stage when other CNs originating from the brainstem also are affected. Therefore, complete neurologic examination should form part of the standard diagnostic approach to dogs presenting with chronic, nonspecific clinical signs consistent with JFS (e.g., coughing, retching, dysphagia, dysphonia), and advanced imaging of the head should be considered. Causes of JFS may be intracranial (e.g., meningioma) or extracranial (e.g., thyroid or carotid body neoplasia). Although MRI remains superior for assessing lesions involving the JF, CT also is likely to provide a diagnostic result. Increased awareness of the clinical signs of JFS and earlier detection of the underlying lesion may lead to earlier treatment and improved outcomes for dogs with JFS, a condition for which the long‐term prognosis is poor.

## Disclosure

Authors declare no off‐label use of antimicrobials.

## Ethics Statement

Approval for the study was granted by the Royal (Dick) School of Veterinary Studies Veterinary Ethical Review Committee: 8.23. Authors declare human ethics approval was not needed.

## Conflicts of Interest

The authors declare no conflicts of interest.

## Supporting information


**Data S1.** Embeded video.

**Table S1.** Table of advanced imaging features.

**Table S2.** Signalment and presentation.

**Table S3.** Neurological examination findings.

**Table S4.** Clinicopathological findings.

**Table S5.** Magnetic resonance imaging (MRI) findings.

**Table S6.** Computed tomography (CT) imaging findings.

**Table S7.** Diagnosis, treatment, outcome.

**Data S2.** Video transcription.

## References

[1] M. H. Rivers , H. J. Svien , and H. L. Baker , “Diagnostic Principles in the Jugular Foramen Syndrome,” Surgical Clinics of North America 43, no. 4 (1963): 1129–1133, 10.1016/S0039-6109(16)37048-7. 14049829

[2] A. De Lahunta , E. Glass , and M. Kent , Veterinary Neuroanatomy and Clinical Neurology, 5th ed. (Saunders, 2021).

[3] J. Das and Y. Al Khalili , “Jugular Foramen Syndrome,” in StatPearls (StatPearls Publishing, 2024), https://www.ncbi.nlm.nih.gov/books/NBK549871/. 31751061

[4] T. J. Vogl and S. Bisdas , “Differential Diagnosis of Jugular Foramen Lesions,” Skull Base 19, no. 1 (2009): 3–16. 19568338 10.1055/s-0028-1103121 PMC2637573

[5] J. Fayad , B. Keles , and E. Derald , “Jugular Foramen Tumors: Clinical Characteristics and Treatment Outcomes,” Otology & Neurotology 31, no. 2 (2010): 299–305, 10.1097/MAO.0b013e3181be6495. 19779386

[6] C. Ruaux , C. P. Champion , S. Pemberton , and J. S. Munday , “Vernet's Syndrome (Jugular Foramen Syndrome) Secondary to Osteoma of the Tympanic Bulla in a Young Male Dog,” Veterinary Record Case Reports 8, no. 3 (2020): e001122, 10.1136/vetreccr-2020-001122.

[7] B. Lluesma , N. T. Whitley , and J. R. Hughes , “Computed Tomographic Features of Canine Intracranial and Jugular Foraminal Masses Involving the Combined Glossopharyngeal, Vagus, and Accessory Nerve Roots,” Veterinary Radiology & Ultrasound 65, no. 3 (2024): 308–316, 10.1111/vru.13359. 38549218

[8] B. K. Sturges , P. J. Dickinson , A. W. Bollen , et al., “Magnetic Resonance Imaging and Histological Classification of Intracranial Meningiomas in 112 Dogs,” Journal of Veterinary Internal Medicine 22, no. 3 (2008): 586–595, 10.1111/j.1939-1676.2008.00042.x. 18466258

[9] J. P. Graham , S. M. Newell , A. K. Voges , G. D. Roberts , and J. M. Harrison , “The Dural Tail Sign in the Diagnosis of Meningiomas,” Vet Radiol Htmlent Glyphamp Asciiamp Ultrasound 39, no. 4 (1998): 297–302, 10.1111/j.1740-8261.1998.tb01609.x. 9710130

[10] W. Mai , Diagnostic MRI in Dogs and Cats (CRC Press, Taylor & Francis Group, 2018).

[11] A. Davies , D. Kidd , S. Stone , and J. MacMahon , “Pharyngeal Sensation and Gag Reflex in Healthy Subjects,” Lancet 345 (1995): 487–488. 7861875 10.1016/s0140-6736(95)90584-7

[12] E. S. Wallace , M. l. Huckabee , and P. Macrae , “Cough Reflex Testing in Clinical Dysphagia Practice,” Advances in Communication and Swallowing 25, no. 2 (2022): 73–81, 10.3233/ACS-220008.

[13] A. B. Erman , A. E. Kejner , N. D. Hogikyan , and E. Feldman , “Disorders of Cranial Nerves IX and X,” Seminars in Neurology 29, no. 1 (2009): 85–92, 10.1055/s-0028-1124027. 19214937 PMC4239699

[14] B. Bordoni , R. R. Reed , P. Tadi , and M. Varacello , “Neuroanatomy, Cranial Nerve 11 (Accessory),” in StatPearls (StatPearls Publishing, 2023). 29939544

[15] H. E. Evans , A. De Lahunta , and M. E. Miller , Miller's Anatomy of the Dog (Elsevier Suanders, 2013).

[16] V. Aige‐Gil , Neuroanatomy of the Dog (Linus Learning, 2022).

[17] S. Manjila , T. Bazil , M. Kay , U. K. Udayasankar , and M. Semaan , “Jugular Bulb and Skull Base Pathologies: Proposal for a Novel Classification System for Jugular Bulb Positions and Microsurgical Implications,” Neurosurgical Focus 45, no. 1 (2018): E5. 10.3171/2018.5.FOCUS18106 29961385

[18] R. J. de Fernanz‐ Thomas and O. De Jesus , “Glomus Jugulare,” in StatPearls (StatPearls Publishing, 2024). 32809324

[19] T. B. Molony , D. E. Brackmann , and W. W. M. Lo , “Meningiomas of the Jugular Foramen,” Otolaryngology–Head and Neck Surgery 106, no. 2 (1992): 128–136, 10.1177/019459989210600202. 1738543

[20] A. Szymanska , M. Szymanski , E. Czekajska‐Chehab , and M. Szczerbo‐Trojanoswka , “Non‐Paraganglioma Tumors of the Jugular Foramen—Growth Patterns, Radiological Presentation, Differential Diagnosis,” Neurologia i Neurochirurgia Polska 49, no. 3 (2015): 156–163. 26048603 10.1016/j.pjnns.2015.04.003

[21] C. Rayappa , “Jugular Foramen Tumors,” An International Journal of Otorhinolaryngology Clinics 3, no. 1 (2011): 15–23, 10.5005/jp-journals-10003-1050.

[22] A. L. Rhoton, Jr. , “The Posterior Fossa Cisterns,” Neurosurgical Focus 47, no. 3 (2000): S287–S297. 10.1097/00006123-200009001-00029 10983312

[23] F. Corrivetti , F. Fraschetti , G. Cacciotti , C. Bernardi , A. Sufianov , and L. Mastronardi , “Inflammatory Pseudotumor Simulating a Jugular Foramen Meningioma: Case Report, Technical Video, and Literature Review,” World Neurosurgery 161 (2022): 106–109, 10.1016/j.wneu.2022.01.069. 35092811

[24] V. F. H. Chong and Y. F. Fan , “Jugular Foramen Involvement in Nasopharyngeal Carcinoma,” Journal of Laryngology and Otology 110, no. 10 (1996): 987–990. 8977870 10.1017/s0022215100135534

[25] I. V. Renuka , P. Premalatha , T. Rayapa Reddy , and D. Rajasekhar , “Clear Cell Carcinoma of Salivary Gland With Intracranial Extension Through Jugular Foramen,” Indian Journal of Otolaryngology and Head & Neck Surgery 67, no. 4 (2015): 422–424, 10.1007/s12070-015-0844-5. 26693463 PMC4678262

[26] F. Laigle‐Donadey , S. Taillibert , and N. Martin‐Duverneuil , “Skull‐Base Metastases,” Journal of Neuro‐Oncology 75 (2005): 63–69. 16215817 10.1007/s11060-004-8099-0

[27] M. Kent , S. A. Arnold , M. Perlini , E. N. Glass , and R. M. Barber , “Unilateral Laryngeal Paralysis Secondary to Otitis Media/Interna in Two Cats,” Journal of the American Animal Hospital Association 58, no. 1 (2022): 42–47, 10.5326/JAAHA-MS-7099.

[28] D. J. Kang , W. K. Park , S. Y. Kim , D. H. Shin , H. M. Park , and M. H. Kang , “Case Report: Villaret's Syndrome Caused by Middle Ear Adenocarcinoma in a Cat,” Frontiers in Veterinary Science 10 (2023): 1225567, 10.3389/fvets.2023.1225567. 37576831 PMC10413872

[29] C. Dewey and R. C. da Costa , Practical Guide to Canine and Feline Neurology (Wiley, 2016).

