# Supplementary material for: Jugular Foramen Syndrome: Concurrent Neurological Deficits, Advanced Imaging Findings, Underlying Diagnoses, and Outcomes in 14 Dogs (2016–2024)
Source: J Vet Intern Med. 2025 Apr 29;39(3):e70088. doi: 10.1111/jvim.70088 (PMC12038936; doi:10.1111/jvim.70088)
Supplement: Supplementary file 2 — Table S1. Table of advanced imaging features. [file JVIM-39-e70088-s002.docx]

**Supplementary Information S1: Table of advanced imaging features.**

| Advanced imaging features | Recorded characteristic |
| --- | --- |
| Modality | CT, MRI or both  Study regions included (i.e. head, neck, thorax, abdomen) |
| Bony changes | Involvement of the jugular foramen  Widening of the jugular foramen  Widening of the tympano-occipital fissure  Widening of condylar canal  Widening of the petro-occipital canal  Widening of the carotid canal  Indentation of the dorsal margin of the tympanic bulla  Osteolysis of the petro-occipital canal  Osteolysis of the tympanic bulla, inner ear structures and adjacent temporal bone  Sclerosis of the petrosal part of the temporal bone  Widening of the hypoglossal canal  Hyperostosis of petrous temporal bone  Sclerosis or thinning of petrous portion of the temporal bone  Sclerosis or thinning of the tympanic portion of the temporal bone  Sclerosis or thinning of basioccipital bone |
| Soft tissue changes | Involvement of the longus capitis, digastricus or laryngeal musculature  Surrounding the internal carotid artery  Nasopharyngeal compression  Trapezius atrophy (cervical and thoracic portions)  Cleidocephalicus atrophy (mastoid and cervical portions)  Sternocephalicus atrophy (mastoid and occipital portions)  Omotransverse muscle atrophy  Laryngeal muscle atrophy  Other muscle atrophy (e.g. temporalis, digastricus, tongue muscles)  Presence of middle ear effusion  Intravascular invasion  Contrast-enhancing material within the hypoglossal canal  Contrast-enhancing material within the carotid canal  Otitis media and externa  Laryngeal displacement |
| Neural structures | Thickening and/or contrast enhancement of cranial nerves other than CNs IX, X and XI  Presence or absence of mass effect (compression of brainstem/cerebellum)  Presence or absence or perilesional oedema  Evidence of raised intracranial pressure (e.g. loss of T2W signal within the cerebral sulci)  Ventricular system assessment (including ventriculomegaly, olfactory recesses, periventricular oedema)  Meningeal contrast enhancement |
| Lesion characteristics | Number of lesions (focal, multifocal, diffuse)  Location and lateralization (intracranial versus extracranial, intra-axial versus extra-axial, left versus right)  Signal intensity on T1W/FLAIR/T2W sequences (MRI) or hyper/iso/hypo-attenuating (CT)  Presence or absence of contrast enhancement, including degree (mild, moderate, marked) and pattern (heterogeneous, homogenous or ring enhancement)  Morphology (plaque-like, cystic, ovoid)  Presence or absence of a dural tail  Involvement of the following anatomical compartments: intracranial, intraforaminal, intrafissural, extracranial |

Abbreviations: CT computed tomography; MRI magnetic resonance imaging; CN cranial nerve; T2W T2-weighted; T1W T1-weighted.
