# Supplementary material for: Jugular Foramen Syndrome: Concurrent Neurological Deficits, Advanced Imaging Findings, Underlying Diagnoses, and Outcomes in 14 Dogs (2016–2024)
Source: J Vet Intern Med. 2025 Apr 29;39(3):e70088. doi: 10.1111/jvim.70088 (PMC12038936; doi:10.1111/jvim.70088)
Supplement: Supplementary file 3 — Table S2. Signalment and presentation. [file JVIM-39-e70088-s005.docx]

**Supplementary Information S2: Signalment and presentation.**

| Case | Signalment | Duration of clinical signs (days) | Presenting complaints | Concurrent medical conditions | Physical examination findings |
| --- | --- | --- | --- | --- | --- |
| 1 | 9.6y MN Lhasa Apso | Chronic (30d) | Sneezing; reverse sneezing; excessive licking; hypersalivation; regurgitation; retching; pica |  | NAD. |
| 2 | 9.9y FN Boxer | Chronic (270d) | Coughing; head tilt; ataxia; knuckling |  | Dry cough; laryngeal stridor; unilateral laryngeal paralysis on induction for GA. |
| 3 | 11y FN SBT | Acute (5d) | Regurgitation; retching; coughing; unilateral temporal muscle atrophy. | Degenerative myelopathy; DISH (diagnosed following investigations). | Palpable soft tissue mass in right jugular furrow. |
| 4 | 14.5y MN Sussex Spaniel | Acute (5d) | Coughing; ataxia; lethargy; head and body turn.  Previous surgery for laryngeal paralysis 2-year prior to presentation. |  | Laryngeal stridor. |
| 5 | 10.6y FN Pug crossbreed | Chronic (210d) | Retching; ventral cervical mass |  | Laryngeal stridor; palpable soft tissue mass in left ventral cervical region; L-sided xeromycertia. |
| 6 | 7.9y FE Irish Setter | Acute (5d) | Head tilt; ataxia; pain opening mouth |  | Pain opening mouth |
| 7 | 8.9y FE WHWT | Chronic (720d) | Dysphonia; regurgitation; coughing; pica |  | Grade I/VI systolic murmur |
| 8 | 9.8y FN Labrador | Chronic (15d) | Head tilt; ataxia |  | NAD |
| 9 | 12.1y MN Soft Coated Wheaten Terrier | Chronic (90d) | Sneezing; retching; panting during exercise; coughing |  | Gagging/retching following tracheal pinch test |
| 10 | 10y MN SBT | Chronic (180d) | Dysphagia; regurgitation; unilateral facial paralysis; head tilt; lethargy |  | Saliva accumulation in left side of mouth; left corneal ulceration; L-sided KCS and xeromycertia. |
| 11 | 10.9y FN WHWT | Chronic (180d) | Lethargy; PU/PD; vacant/collapse episodes | Hyperadrenocorticism (diagnosed prior to presentation). | Unilateral oral saliva accumulation in the R side of the mouth; prescapular and submandibular lymphadenomegaly; R-sided xeromycertia. |
| 12 | 9.6y FN Springer Spaniel | Chronic (210d) | Sneezing; retching; coughing |  | laryngeal stridor; coughing; muscle atrophy on left side of neck; unilateral laryngeal paralysis on induction of GA. |
| 13 | 9.1y FN French Bulldog | Chronic (35d) | Dysphagia; retching; unilateral facial paralysis; Horner’s syndrome | C5-6 IVDE (diagnosed following investigations). | Right thoracic limb lameness; unilateral laryngeal paralysis on induction of GA. |
| 14 | 10.75y FN SBT | Chronic (210d) | Dysphonia; coughing; unilateral muscle (tongue) atrophy; head tilt; facial twitching | Previous surgery for T11/12 IVDP (diagnosed prior to presentation). | NAD; Unilateral laryngeal paralysis on induction of GA. |

Abbreviations: SBT Staffordshire bull terrier; WHWT west highland white terrier; FN female neutered; MN male neutered; FE female entire; URT upper respiratory tract; DISH diffuse idiopathic skeletal hyperostosis; KCS keratoconjunctivitis sicca; NAD no abnormalities detected; PU/PD polyuria/polydipsia; IVDE intervertebral disc extrusion; IVDP intervertebral disc protrusion; R right; L left; GA general anaesthetic.
