# Supplementary material for: Jugular Foramen Syndrome: Concurrent Neurological Deficits, Advanced Imaging Findings, Underlying Diagnoses, and Outcomes in 14 Dogs (2016–2024)
Source: J Vet Intern Med. 2025 Apr 29;39(3):e70088. doi: 10.1111/jvim.70088 (PMC12038936; doi:10.1111/jvim.70088)
Supplement: Supplementary file 4 — Table S3. Neurological examination findings. [file JVIM-39-e70088-s003.docx]

**Supplementary Information S3: Neurological examination findings**

| Case | Neurological examination findings | Neurolocalization | Neurolocalization taking history/PE into consideration | Lateralization | Final diagnosis |
| --- | --- | --- | --- | --- | --- |
| 1 | Equivocal L-sided head tilt and reduced gag reflex. | Cranial polyneuropathy involving CNs VIII, IX and X [central or peripheral portions]. | Consider involvement of CNs IX and X (retching and regurgitation) | Left | Left cerebellomedullary plaque-like extra-axial mass - suspected **meningioma.** |
| 2 | L-sided head tilt; ambulatory PL proprioceptive ataxia with monoparesis and spontaneous knuckling of R PL; postural reaction deficits in R PL; reduced patellar reflex L PL; mild lumbar hyperaesthesia. | **Multifocal:** L peripheral vestibular system  + R T3-L3 spinal cord segments. | Consider involvement of CN X (URT stertor) | Left | Left cerebellomedullary plaque-like extra-axial mass - suspected **meningioma.**  *[+ concurrent and unrelated degenerative myelopathy)* |
| 3 | R-sided Horner’s syndrome; R-sided temporalis muscle atrophy. | **Multifocal**: sympathetic innervation to R eye (first, second or third order neuron) and the temporalis muscle, mandibular branch of CN V or neuromuscular junction. | Consider involvement of CNs IX and X (retching and regurgitation) | Right | Right-sided ventral cervico-occipital mass - suspected **thyroid carcinoma.** |
| 4 | Obtundation; R-sided head and body turn; non-ambulatory tetraparesis; postural reaction deficits in R TL and PL; L-sided spontaneous ventral strabismus; intermittent spontaneous horizontal nystagmus. | **Central vestibular system (brainstem)**. | Consider involvement of CN X (URT stertor) | Right | Right cerebellomedullary angle ovoid extra-axial mass - **suspected** **meningioma.** |
| 5 | Normal | N/A | Consider involvement of CNs IX and X (retching and respiratory stertor) | N/A | Left retropharyngeal and ventral cervico-occipital mass – **confirmed** **compact follicular thyroid carcinoma.** |
| 6 | R-sided head tilt and vestibular ataxia. | Peripheral vestibular system. |  | Right | **Right cholesteatoma, para-aural abscess,** otitis externa, media and interna |
| 7 | Normal | N/A | Consider involvement of CN X (dysphonia and regurgitation) | N/A | Left cerebellomedullary plaque-like extra-axial mass- **suspected meningioma.** |
| 8 | R-sided head tilt; non-ambulatory tetraparesis with vestibular ataxia; postural reaction deficits in R TL and PL; rotary jerk conjugate nystagmus with fast phase to the L. | **Central vestibular system (brainstem)** |  | Right | Right extra-axial ovoid cerebellopontine angle mass- **confirmed** **mixed/transitional grade 1 meningioma.** |
| 9 | Normal | N/A | Consider involvement of CNs IX and X (retching and coughing) | N/A | Left cerebellomedullary plaque-like extra-axial mass – **suspected** **meningioma.** |
| 10 | Mild L-sided head tilt; L facial nerve paralysis; L-sided tongue atrophy and deviation to the L; transient positional spontaneous horizontal/rotatory jerk nystagmus with fast phase to R; L KCS and xeromycteria. | Cranial polyneuropathy involving CNs VII (motor and parasympathetic fibers), VIII and XII [central or peripheral portions]. | Consider involvement of CNs IX and X (dysphagia and regurgitation) | Left | Left extra-axial plaque-like cerebellopontine angle mass - **suspected meningioma.** |
| 11 | Equivocal R-sided head tilt; mild vestibular ataxia; R-sided tongue atrophy; R-sided xeromycertia. | Cranial polyneuropathy involving CNs VIII and XII [central or peripheral portions]. | Consider involvement of CNs IX and X (saliva accumulation) and VII (xeromycertia) | Right | Right extra-axial plaque-like cerebellopontine angle mass – **suspected** **meningioma.** |
| 12 | Unilateral cervical muscle atrophy; query L-sided tongue paresis. | Cranial polyneuropathy involving CNs XI + XII [central or peripheral portions]. | Consider involvement of CNs IX and X (retching, coughing). | Left | Left extra-axial cerebellopontine angle cystic mass- **suspected meningioma.** |
| 13 | Proprioceptive ataxia affecting PLs with R TL lameness; postural reaction deficits in L PL; muscle atrophy L PL; cutaneous trunci cut off at the level of the L TLJ; L-sided tongue atrophy; subjective neck pain. | **Multifocal:** CN XII + T3-L3 spinal cord segments (left lateralising) + cervical region (including soft tissues, vertebrae, intervertebral discs, articular facet joints, nerve roots and meninges) | Consider involvement of CNs IX and X (dysphagia, retching) | Left | Left retropharyngeal ventral cervico-occipital mass - **suspected** **carotid body paraganglioma.**  [+ concurrent IVDD] |
| 14 | Mild L-sided head tilt; vestibular ataxia; postural reaction deficits in L PL; reduced gag reflex; L-sided tongue paresis and atrophy. | Cranial polyneuropathy involving CNs VIII, IX and X and XII [central or peripheral portions].  + T3-L3 spinal cord segments (L lateralising) | Consider involvement of CN VII (unilateral facial myokymia). | Left | Left extra-axial plaque-like cerebellopontine angle mass -**suspected meningioma.**  [+ previous corpectomy at T11-12 for IVDP] |

*Abbreviations: PE physical examination; PL pelvic limb; TL thoracic limb; R right; L left; CN cranial nerve; KCS keratoconjunctivitis sicca; URT upper respiratory tract; PNST peripheral nerve sheath tumour; IVDD intervertebral disc disease; IVDP intervertebral disc protrusion.*
