# Supplementary material for: Jugular Foramen Syndrome: Concurrent Neurological Deficits, Advanced Imaging Findings, Underlying Diagnoses, and Outcomes in 14 Dogs (2016–2024)
Source: J Vet Intern Med. 2025 Apr 29;39(3):e70088. doi: 10.1111/jvim.70088 (PMC12038936; doi:10.1111/jvim.70088)
Supplement: Supplementary file 5 — Table S4. Clinicopathological findings. [file JVIM-39-e70088-s004.docx]

**Supplementary Information S4: Clinicopathological findings.**

| Case | Haematology, biochemistry, electrolytes and blood gas analysis | Urinalysis | CSF analysis | Cytology/histology | Other |
| --- | --- | --- | --- | --- | --- |
| 1 | NAD | N/A | N/A |  | Nasopharyngeal and upper GI endoscopy normal. |
| 2 | Hypercholesterolaemia (10.08mmol/L, RI: 3.2-6mmol/L) | N/A | N/A |  | T4/TSH normal.  SOD1 homozygous. |
| 3 | Hypercholesterolaemia (7.37mmol/L, RI: 3.2-6mmol/L); elevated total bilirubin (4.7umol/L, RI: 0.1-4.2umol/L) | N/A | N/A | Cytology R laryngeal mass: epithelial carcinoma. |  |
| 4 | NAD | N/A | Cervical: TNCC 540cells/ul; RBC 7/ul; cytology: neutrophilic pleocytosis (90% non-degenerative neutrophils, 2% small lymphocytes, 8% large mononuclear cells). |  |  |
| 5 | Hypocholesterolaemia (2.66mmol/L, RI: 3.2-6mmol/L); elevated ALKP (139U/L, RI: 0-130U/L) | Dipstick: protein 3+, trace blood, bilirubin 1+  UPC 1.21 | N/A | Cytology L tonsil: reactive changes.  Cytology L cervical mass: consistent with thyroid tumour.  Histology (tru-cut) L cervical mass: compact-follicular thyroid carcinoma. |  |
| 6 | Elevated ALKP (159U/L, RI: 0-130U/L); hypercholesterolaemia (8.7mmol/L, RI: 3.2-6.2mmol/L) | N/A | N/A | Cytology:  External ear canals: Malassezia spp.  Left middle ear fluid: Malassezia ++, neutrophils +.  Right middle ear fluid: cocci (chains) ++, degenerate neutrophils ++++.  FNA of retropharyngeal mass: Neutrophilic inflammation.  FNA of right medial retropharyngeal lymph node: markedly reactive node with evidence of extramedullary hematopoiesis; possible neutrophilic inflammation.  Histopathology (surgical sample): cholesteatoma. | Bacterial culture and sensitivity (left and right middle ear aspirate): Enterococcus faecalis and Malassezia sp. |
| 7 | NAD | N/A | N/A |  |  |
| 8 | Mild hypoalbuminaemia (25.6g/L, RI: 26.3-38.2g/L); mild hypoglobulinaemia (19.6g/L, RI: 23.4-42.2g/L); mild elevation in ALT (230.8U/L, RI:19.8-124U/L) and ALKP (417U/L, RI: 0-130U/L); moderate elevation in lipase (9540U/L, RI:66-1265U/L) and amylase (2803.9, RI: 100-1200U/L).  Blood gas: mild hypokalaemia (3.4mmol/L, RI: 3.6-4.8mmol/L) and marked hyperlactatemia (9.8mmol/L, I 0.6-2.5mmol/L) | N/A | N/A | Histopathology (PM): Mixed/transitional meningioma; grade 1 | Hypocobalaminaemia (223ng/L, cut off >400ng/L for enteropathy)  AUS: peri-gastric and pancreatic steatitis; mildly enlarged and nodular liver (steroid hepatopathy suspected); heterogenous spleen (EMH suspected); gall bladder sediment and mineralisation. |
| 9 | NAD | NAD | N/A |  | Faecal analysis: normal. |
| 10 | NAD | N/A | N/A |  |  |
| 11 | Thrombocytosis and elevated ALKP | Protein ++++ on dipstick | Lumbar: albuminocytological dissociation (TNCC 2.2/ul, RI: 0-5/ul); TP 75mg/dL, RI: <25mg/dL) | Cytology R prescapular lymph node: mild reactive lymphoid hyperplasia. |  |
| 12 | Mild hypercholesterolaemia  Mild anaemia | N/A | N/A |  | Angiodetect SNAPtest: negative. |
| 13 | NAD | N/A | N/A | Gross PM: suspected carotid body neoplasia (absence of histopathology) |  |
| 14 | Hypercholesterolemia (10.99mmol/l, RI: 2-8mmol/L); elevated ALKP (294.61U/L, RI: 0-230U/L); mild elevation in GLDH (10.62, 0-10U/L); mild hyperglobulinaemia (45.5, 26-44g/L) | N/A | N/A |  | T4/TSH: WNL. |

Abbreviations: CSF cerebrospinal fluid; NAD no abnormalities detected; N/A not applicable; FNA fine needle aspirate; GI gastrointestinal; RI reference interval; T4/TSH thyroid hormone/thyroid stimulating hormone; SOD1 superoxide dismutase 1; R right; L left; TNCC total nucleated cell count; TP total protein; RBC red blood cells; ALKP alkaline phosphatase; UPC urine protein:creatinine ratio; ALT alanine aminotransferase; PM post mortem; GLDH glutamate dehydrogenase; WNL within normal limits.
