# Supplementary material for: Jugular Foramen Syndrome: Concurrent Neurological Deficits, Advanced Imaging Findings, Underlying Diagnoses, and Outcomes in 14 Dogs (2016–2024)
Source: J Vet Intern Med. 2025 Apr 29;39(3):e70088. doi: 10.1111/jvim.70088 (PMC12038936; doi:10.1111/jvim.70088)
Supplement: Supplementary file 6 — Table S5. Magnetic resonance imaging (MRI) findings. [file JVIM-39-e70088-s007.docx]

**Supplementary information S5: Magnetic Resonance Imaging (MRI) findings.**

| Case | | Main finding and diagnosis | | Bony changes | | Soft tissue changes | | Neural structures | | Sequence characteristics *(relative to the surrounding grey matter)* | | Location (intracranial, intra-foraminal, intra-fissural, extracranial) | |
| --- | --- | --- | --- | --- | --- | --- | --- | --- | --- | --- | --- | --- | --- |
| INTRACRANIAL ORIGIN | | | | | | | | | | | | | |
| 2 | | Left cerebellomedullary plaque-like extra-axial mass.  Diagnosis: suspected **meningioma.** | | - Widening of JF - Widening of TO fissure - Sclerosis of petrous portion of the temporal bone. | | - CE extending through the hypoglossal canal - Left temporalis muscle atrophy. | | - Mass effect - Dural tail. | | - T2W/FLAIR hyperintense - T1W hypointense - Strong homogeneous CE. | | - Intracranial - Intraforaminal - Intrafissural | |
| 4 | | Right cerebellomedullary angle extra-axial ovoid mass.  Diagnosis: suspected **meningioma.** | | - Widening of JF - Widening of TO fissure - Indentation of dorsal margin TB. | | - None. | | - Mass effect - Perilesional oedema - Dural tail. | | - T2W/FLAIR hyperintense - T1W hypointense - Strong heterogeneous CE | | - Intracranial - Intraforaminal - Intrafissural | |
| 8 | | Right extra-axial cerebellopontine angle ovoid mass.  Diagnosis: **confirmed** **mixed/transitional grade 1 meningioma.** | | - Widening of JF - Widening of TO fissure - Indentation of dorsal margin TB. | | - None. | | - Mass effect - Perilesional oedema - CNVIII thickening and CE +/- equivocal CN VII CE - Dural tail. | | - T2W hyperintense - FLAIR isointense - T1W iso- to hypo-intense - Strong slightly heterogeneous CE. | | - Intracranial - Intraforaminal - Intrafissural | |
| 10 | | Left extra-axial plaque-like cerebellopontine angle mass.  Diagnosis: **suspected meningioma.** | | - Widening of JF. | | - None. | | - Mass effect - CN VII and CNVIII thickening and CE - Dural tail sign - CN XII thickening and CE. | | - T2W/FLAIR hyperintense - T1W hypointense - Strong homogeneous CE. | | - Intracranial - Intraforaminal - Intrafissural | |
| 11* | | Right extra-axial plaque like cerebellopontine angle mass.  Diagnosis: **suspected meningioma.** | | - Widening of JF. - Widening of TO fissure - Widening of hypoglossal canal. | | - Caudal digastricus muscle atrophy. | | - Mass effect - Perilesional oedema - CN V, VII, VIII and CN XII thickening and CE - Raised ICP - Obstructive hydrocephalus - Dural tail sign. | | - Heterogeneously T2/FLAIR hyperintense - T1W mixed hyper- to hypo-intense - Strong homogenous CE. | | - Intracranial - Intraforaminal - Intrafissural | |
| 12* | | Left extra-axial cerebellopontine angle cystic mass.  Diagnosis: **suspected meningioma.** | | - Widening of JF - Widening of TO fissure - Widening of hypoglossal canal. | | - Surrounds internal carotid artery - CE extending through the hypoglossal canal. | | - Mass effect - Perilesional oedema - CN VII, CN VIII and CN XII enlargement and CE - Dural tail sign | | - T2W/FLAIR hyperintense, T1W hypointense, non-CE cystic component - T2W/FLAIR hyperintense, T1W isointense and strong homogeneously CE border. | | - Intracranial - Intraforaminal - Intrafissural | |
| 14* | | Left extra-axial plaque-like cerebellopontine angle mass.  Diagnosis: **suspected meningioma.** | | - Widening of the JF - Widening of the TO fissure. | | - CE within the hypoglossal canal - Atrophy of the mastoid and occipital portion of the sternocephalicus - Left caudal digastricus muscle atrophy - Left tongue atrophy. | | - CN V, VII and VIII CE - Dural tail sign - Mass effect. | | - T2W/FLAIR mildly hyperintense - T1W iso- to mildly hypo-intense - strongly, homogeneous CE. | | - Intracranial - Intraforaminal - Intrafissural | |
| EXTRACRANIAL ORIGIN | | | | | | | | | | | | | |
| 3* | | Right-sided ventral cervico-occipital mass.  Diagnosis: suspected **thyroid carcinoma.** | | - Widening of TO fissure - Widening of condylar canal. | | - Involvement of longus capitis muscle - Surrounding the internal carotid artery - Nasopharyngeal compression - Mild right-sided temporalis muscle atrophy | | - Dilation of the internal carotid a. - Meningeal CE - Mild asymmetrical R-sided CN V CE. | | - Signal void on T2* (salt and pepper appearance) - Heterogeneously T2W hyperintense - T1W iso- and hyper-intense - Markedly heterogeneous CE. | | - Extracranial - Intraforaminal - Intrafissural - Intracranial | |

Abbreviations: JF jugular foramen; TO tympano-occipital; TB tympanic bulla; CE contrast-enhancing/enhancement; CN cranial nerve.

*CT study also performed
