# Supplementary material for: Jugular Foramen Syndrome: Concurrent Neurological Deficits, Advanced Imaging Findings, Underlying Diagnoses, and Outcomes in 14 Dogs (2016–2024)
Source: J Vet Intern Med. 2025 Apr 29;39(3):e70088. doi: 10.1111/jvim.70088 (PMC12038936; doi:10.1111/jvim.70088)
Supplement: Supplementary file 7 — Table S6. Computed tomography (CT) imaging findings. [file JVIM-39-e70088-s001.docx]

**Supplementary Information S6: Computed tomography (CT) imaging findings.**

| Case | | Study regions | Main finding and diagnosis | Bony changes | Soft tissue changes | Neural structures | Signal properties | Location (intracranial, intra-foraminal, intra-fissural, extracranial) |
| --- | --- | --- | --- | --- | --- | --- | --- | --- |
| INTRACRANIAL ORIGIN | | | | | | | | |
| 1 | Head (to C2) + Thorax (including C5 caudal) | | Left cerebellomedullary plaque-like extra-axial mass.  Diagnosis: **suspected** **meningioma.** | - Widening of JF - Widening of TO fissure - Indentation of dorsal margin of TB - Widening of hypoglossal canal. | - Soft tissue attenuating and CE material within the widened JF.   Muscle atrophy (unilateral, left):   - Trapezius: cervical (y), thoracic (y). - Cleidocephalicus:   mastoid (y), cervical (y).   - Sternocephalicus: mastoid (y), occipital (y). - Omotransverse: (N/A). - Laryngeal (y). | - Mass effect - Perilesional oedema - Dural tail | - Soft tissue (iso-) attenuating - Strong homogeneous CE. | - Intracranial - Intraforaminal - Intrafissural |
| 7 | Head + Neck + Thorax | | Left cerebellomedullary plaque-like extra-axial mass.  Diagnosis: **suspected meningioma.** | - Widening of JF - Widening of TO fissure - Indentation of dorsal margin of TB - Widening of hypoglossal canal | - Soft tissue attenuating and CE material within the widened JF. - CE extending into the hypoglossal canal and carotid canal.   Muscle atrophy (unilateral, left):   - Trapezius: cervical (y), thoracic (y). - Cleidocephalicus: - mastoid (y), cervical (y) - Sternocephalicus: mastoid (y), occipital (y). - Omotransverse: (n). | - Mass effect. | - Soft tissue (iso-) attenuating - Strong homogeneous CE. | - Intracranial - Intraforaminal - Intrafissural |
| 9 | Head + Neck + Thorax | | Left cerebellomedullary mass plaque-like extra-axial mass.  Diagnosis: suspected **meningioma.** | - Sclerosis of petrous temporal bone - Hyperostosis of petrous temporal bone. | Soft tissue attenuating and CE material within the JF.  Muscle atrophy (unilateral, left):   - Trapezius: cervical (y), thoracic (n) - Cleidocephalicus: mastoid (y), cervical (y) - Sternocephalicus: mastoid (y), occipital y) - Omotransverse (n), | - Mass effect. | - Soft tissue (iso-) attenuating - Strong heterogeneous CE. | - Intracranial - Intraforaminal - Intrafissural, |
| 11* | Head (up to C2-3) | | Right extra-axial plaque-like cerebellopontine angle mass.  Diagnosis: **suspected meningioma.** | - Widening of JF - Widening of the TO fissure - Thinning of petrous temporal bone - Widening of hypoglossal canal - Sclerosis of basioccipital bone. | - Soft tissue attenuating and CE material within the widened JF.   Muscle atrophy (unilateral, right):   - Trapezius: cervical (N/A), thoracic (N/A) - Cleidocephalicus: mastoid (y), cervical (y) - Sternocephalicus: mastoid (y), occipital (y) - Omotransverse (y). | - Mass effect. | - Soft tissue (iso-) attenuating - Strongly CE | - Intracranial - Intraforaminal - Intrafissural |
| 12* | Head + neck + thorax + abdomen. | | Left extra-axial cerebellopontine angle cystic mass.  Diagnosis: **suspected meningioma.** | - Widening of JF - Widening of TO fissure - Widening of hypoglossal canal - Hyperostosis of petrous temporal bone. | - Soft tissue attenuating and CE material within the widened JF. - Surrounds internal carotid artery.   Muscle atrophy (unilateral, left):   - Trapezius: cervical (N/A), thoracic (N/A). - Cleidocephalicus:mastoid (y), cervical (y). - Sternocephalicus: Mastoid (y), occipital (y). - Omotransvere (y) - Laryngeal (y). | - Mass effect. | - Soft tissue (iso-) attenuating - Strongly CE mass with fluid attenuating and non-CE (cystic) component. | - Intracranial - Intraforaminal - Intrafissural. |
| 14* | Head + neck + thorax + abdomen. | | Left extra-axial plaque-like cerebellopontine angle mass.  Diagnosis: **suspected meningioma**. | - Widening of JF. | - Soft tissue attenuating and CE material within the widened JF. - CE extension through hypoglossal canal.   Muscle atrophy (unilateral, left):   - Trapezius: cervical (y), thoracic (y) - Cleidocephalicus: mastoid (y), cervical (n) - Sternocephalicus: mastoid (y), occipital (y) - Omotransverse (n) - Laryngeal (y) – sternohyoid muscle - Caudal digastricus muscle - Tongue muscle. | - Mass effect | - Soft tissue (iso-) attenuating - Strong heterogenous CE. | - Intracranial - Intraforaminal - Intrafissural. |
| EXTRACRANIAL ORIGIN | | | | | | | | |
| 3* | Head + neck + Thorax + Abdomen | | Right-sided ventral cervico-occipital mass.  Diagnosis: suspected **thyroid carcinoma.** | - Widening of JF - Widening of TO fissure - Widening of the condylar canal - Osteolysis of petro-occipital canal - Thinning of tympanic portion of the temporal bone. | - Soft tissue attenuating and CE material within the widened JF - Nasopharyngeal compression - Involvement of caudal digastricus muscle - Surrounds internal carotid artery and invades carotid canal - CE within the hypoglossal and carotid canal. - CE extending into sternohyoid muscle.   Muscle atrophy (unilateral, right):   - Trapezius: cervical (y), thoracic (y) - Cleidocephalicus: mastoid (y), cervical (y) - Sternocephalicus: mastoid (y), occipital (y) - Omotransverse: (n) - Temporalis muscle. |  | - Soft tissue (iso-) attenuating - Strong and mildly heterogeneous CE. | - Extracranial - Intraforaminal - Intrafissural. |
| 5 | Head (up to C2) + Thorax (including C5-6 caudal) | | Left retropharyngeal and ventral cervico-occipital mass.  Diagnosis: **compact follicular thyroid carcinoma.** | - Widening of JF - Widening of TO fissure - Indentation of dorsal margin of TB - Widening of hypoglossal canal - Lysis of TB. | - Soft tissue attenuating and CE material within the widened JF. - Involvement of longus capitis and digastricus mm. - Displacement of larynx - Extension of CE to hypoglossal canal - Surrounds internal carotid artery - Nasopharyngeal compression.   Muscle atrophy:   - Trapezius: No - Cleidocephalicus: mastoid (n), cervical (n) - Sternocephalicus: mastoid (n), occipital (n) - Omotransverse: (n) | - Extension of CE material into intracranial cavity. | - Soft tissue (iso-) attenuating - Strong heterogeneous CE. | - Extracranial - Intrafissural - Intraforaminal - Intracranial |
| 6 | Head (up to C2) + Thorax (including C4 caudal) | | **Right cholesteatoma, para-aural abscess,** otitis externa, media and interna. | - Widening of JF - Osteolysis of TB and inner ear structures - Osteolysis of petrous portion of the temporal bone - Widening of hypoglossal canal | - Soft tissue attenuating and CE material within the widened JF and expansion into TO fissure - Nasopharyngeal compression - Extension of CE into carotid and hypoglossal canal - Otitis media and externa   Muscle atrophy:   - Trapezius: (n) - Cleidocephalicus: mastoid (n), cervical (n) - Sternocephalicus: mastoid (n), occipital (n) - Omotransverse: (n). | - Extension of CE material into intracranial cavity. | - Soft tissue attenuating with central fluid attenuating regions. - Mild peripheral ring CE. | - Extracranial - Intrafissural - Intraforaminal - Intracranial |
| 13 | Head + entire vertebral column + thoracic limbs. | | Left retropharyngeal ventral cervico-occipital mass.  Diagnosis: **carotid body paraganglioma.** | - Widening of JF - Widening of TO fissure - Indentation of dorsal margin of TB - Widening of hypoglossal canal - Widening of the petro-occipital and carotid canal - Osteolysis of the petro-occipital canal. | - Soft tissue attenuating and CE material within the widened JF. - Surrounds internal carotid artery.   Muscle atrophy (unilateral, left):   - Trapezius: cervical (y), thoracic (y) - Cleidocephalicus: mastoid (y), cervical (y) - Sternocephalicus: mastoid (y), occipital (y). - Omotransverse: (n). | - Extension of CE into the intracranial space and mild meningeal contrast enhancement. | - Soft tissue (iso-) attenuating - Strong heterogeneous CE. | - Extracranial - Intrafissural - Intraforaminal - Intracranial. |

Abbreviations: JF jugular foramen; TO tympano-occipital; TB tympanic bulla; CE contrast-enhancing;

*MRI study also performed
