# Supplementary material for: Jugular Foramen Syndrome: Concurrent Neurological Deficits, Advanced Imaging Findings, Underlying Diagnoses, and Outcomes in 14 Dogs (2016–2024)
Source: J Vet Intern Med. 2025 Apr 29;39(3):e70088. doi: 10.1111/jvim.70088 (PMC12038936; doi:10.1111/jvim.70088)
Supplement: Supplementary file 8 — Table S7. Diagnosis, treatment, outcome. [file JVIM-39-e70088-s009.docx]

**Supplementary Information S7: Diagnosis, Treatment, Outcome**

| Case | Diagnosis | Treatment | Outcome | Follow up (days) |
| --- | --- | --- | --- | --- |
| 1 | Left cerebellomedullary plaque-like extra-axial mass - **suspected** **meningioma.** | **Palliative**: corticosteroids | Lost to further follow up | Lost to further follow up |
| 2 | Left cerebellomedullary plaque-like extra-axial mass - **suspected** **meningioma.** | **Palliative**: corticosteroids | PTS | 16 |
| 3 | Right-sided ventral cervico-occipital mass - suspected **thyroid carcinoma.** | **Palliative**: meloxicam | Lost to further follow up | Lost to further follow up |
| 4 | Right cerebellomedullary angle extra-axial ovoid mass- **suspected** **meningioma.** | N/A | PTS following diagnostic investigations | N/A |
| 5 | Left ventral cervical mass – confirmed **compact follicular thyroid carcinoma** | **Radiation therapy**; imatinib; meloxicam | Alive at last follow up; experiencing paroxysmal episodes of excessive panting which self-resolved (possibly pain related) | 211 |
| 6 | **Right para-aural abscess, cholesteatoma, otitis externa, media and interna** | Surgery (TECA + LBO); antibiotics (amoxicillin clavulanic acid); analgesia (amantadine, paracetamol); omeprazole; topical clotrimazole | Reported to be neurologically normal at recheck appointment at referral institution. | 477 |
| 7 | Left cerebellomedullary plaque-like extra-axial mass - **suspected meningioma** | **Palliative**: corticosteroids | Lost to further follow up | Lost to further follow up |
| 8 | Right extra-axial ovoid cerebellopontine angle mass – confirmed **mixed/transitional meningioma; grade I** | N/A | PTS following diagnostic investigations | N/A |
| 9 | Left cerebellomedullary extra-axial plaque-like mass - **suspected** **meningioma.** | **Palliative**: corticosteroids. | Alive at last follow up | 57 |
| 10 | Right extra-axial plaque like cerebellopontine angle mass ***-* suspected meningioma** | **Chemotherapy**: hydroxyurea | Lost to further follow up | Lost to further follow up |
| 11 | Right extra-axial plaque-like cerebellopontine angle mass - **suspected meningioma.** | **Radiation therapy**; corticosteroids; gabapentin | Alive at last follow up; requiring symptomatic treatment for gagging/hypersalivation. | 347 |
| 12 | Left extra-axial cerebellopontine angle cystic mass -**suspected meningioma.** | **Radiation therapy**; corticosteroids; gabapentin; paracetamol | Alive at last follow up; requiring symptomatic treatment for gagging/hypersalivation. | 218 |
| 13 | Left retropharyngeal mass – **suspected carotid body paraganglioma** | N/A | PTS following diagnostic investigations. | N/A |
| 14 | Left extra-axial plaque-like cerebellopontine angle mass - **suspected meningioma**. | **Radiation therapy**; prednisolone; gabapentin; paracetamol | Alive at last follow up. | 336 |

*Abbreviations: N/A not applicable; PTS put to sleep; TECA total ear canal ablation; VBO ventral bulla osteotomy.*
