# Supplementary material for: Jugular Foramen Syndrome: Concurrent Neurological Deficits, Advanced Imaging Findings, Underlying Diagnoses, and Outcomes in 14 Dogs (2016–2024)
Source: J Vet Intern Med. 2025 Apr 29;39(3):e70088. doi: 10.1111/jvim.70088 (PMC12038936; doi:10.1111/jvim.70088)
Supplement: Supplementary file 9 — Data S2. Video transcription. [file JVIM-39-e70088-s006.docx]

**TRANSCRIPTION**

Semiology of jugular foramen syndrome in dogs:

Dog with plaque-like suspected meningioma

1. Retching
2. Laryngeal stridor
